# Socioeconomic inequalities in birth outcomes: An 11-year analysis in Colombia

**DOI:** 10.1371/journal.pone.0255150

**Published:** 2021-07-29

**Authors:** Carol C. Guarnizo-Herreño, Gabriel Torres, Giancarlo Buitrago

**Affiliations:** 1 Departamento de Salud Colectiva, Facultad de Odontología, Universidad Nacional de Colombia, Bogotá, Colombia; 2 Instituto de Investigaciones Clínicas, Facultad de Medicina, Universidad Nacional de Colombia, Bogotá, Colombia; 3 Hospital Universitario Nacional de Colombia, Bogotá, Colombia; University of Washington, UNITED STATES

## Abstract

**Objective:**

To examine socioeconomic inequalities in birth outcomes among infants born between 2008 and 2018 and assessed trends in inequalities during that period in Colombia, a middle-income country with high levels of inequality emerging from a long internal armed conflict.

**Methods:**

Using birth certificate data in Colombia, we analysed the outcomes of low birth weight, an Apgar score <7 at 5 minutes after birth and the number of prenatal visits among full-term pregnancies. Maternal education and health insurance schemes were used as socioeconomic position (SEP) indicators. Inequalities were estimated using the prevalence/mean of the outcomes across categories of the SEP indicators and calculating the relative and slope indices of inequality (RII and SII, respectively).

**Results:**

Among the 5,433,265 full-term singleton births analysed, there was a slight improvement in the outcomes analysed over the study period (lower low-birth-weight and Apgar<7 prevalence rates and higher number of prenatal visits). We observed a general pattern of social gradients and significant relative (RII) and absolute (SII) inequalities for all outcomes across both SEP indicators. RII and SII estimates with their corresponding CIs revealed a general picture of no significant changes in inequalities over time, with some particular, time-dependent exceptions. When comparing the initial and final years of our study period, inequalities in low birth weight related to maternal education increased while those in Apgar score <7 decreased. Relative inequalities across health insurance schemes increased for the two birth outcomes but decreased for the number of prenatal visits.

**Conclusion:**

The lack of a consistent improvement in the magnitude of inequalities in birth outcomes over an 11-year period is a worrying issue because it could aggravate the cycle of inequality, given the influence of birth outcomes on health, social and economic outcomes throughout the life course. The findings of our analysis emphasize the importance of policies aimed at providing access to quality education and providing a health care system with universal coverage and high levels of integration.

## Introduction

Maternal and birth outcomes such as birth weight and gestational age remain important public health indicators. These outcomes have been shown to influence health and wellbeing not only during childhood but also throughout the life course, having an impact on child development, cardiovascular diseases, all-cause mortality, and different social and economic outcomes in adulthood [1–5]. Birth outcomes are also considered key indicators of population health and social development given that they capture the cumulative effect of different determinants of health and are associated with general wellbeing and access to health care [6, 7]. Therefore, reproductive and birth outcomes are thought to be a sensitive reflection of the socioeconomic and health care conditions of each country.

Adverse birth outcomes (e.g., low birth weight) disproportionately affect low- and middle-income countries [8, 9] and women and children from lower socioeconomic backgrounds across countries [10]. Inequalities in various outcomes, including birth weight, gestational age at birth and neonatal death, have been observed with worse outcomes occurring among those with lower levels of wealth, income, education and occupational social class [11–13]. Maternal education, in particular, has been identified as a key determinant of birth outcomes that acts through various pathways, including access to information, health care utilization, nutrition and health-related behaviours [14]. For example, mothers with lower educational levels tend to have poorer economic conditions and experience greater exposure to stress, which in turn results in a higher likelihood of engaging in risk-taking behaviours such as smoking and alcohol/drug abuse [15]. Less-educated mothers also make, on average, fewer prenatal care visits and are less likely to access skilled birth attendance care during childbirth [16].

Although largely preventable, adverse maternal and birth outcomes remain an important public health policy concern in many countries, including Colombia, where in recent years, reductions in perinatal and maternal deaths have stagnated [17, 18]. Analysing the underlying causes of these issues, including the role of socioeconomic conditions, can make relevant contributions to the evidence base that should inform the design and implementation of public health strategies and public policies more broadly. Moreover, assessing changes in the magnitude of these inequalities over time is a very useful tool for health surveillance since it allows for the measurement of the potential impact of policies and interventions aimed at tackling such inequalities. These issues have barely been explored in Colombia, a country with high levels of inequality where the informal sector plays a key role in the economy and where the health profile is very complex, combining the health consequences of a 50-year-long internal armed conflict, a high burden of both chronic and infectious diseases and problems with access to and the quality of health care [19–22].

Few studies have examined inequalities in birth outcomes in middle-income countries and whether those inequalities have changed over time. Furthermore, to our knowledge, no previous study has quantified the magnitude of socioeconomic inequalities in birth outcomes in Colombia and described its changes over time. Using birth certificate data, our study examined socioeconomic inequalities in birth outcomes among infants born in Colombia between 2008 and 2018 and assessed trends in inequalities during that period. We used maternal education as an indicator of socioeconomic position (SEP), and given the special features of the Colombian health care system, inequalities by health insurance scheme were also examined.

## Methods

### Data source and study population

Our study included all full-term live births (registered in birth certificate records) that occurred in Colombia between 2008 and 2018. Data were obtained from the Colombian National Department of Statistics (DANE), which collects and oversees birth certificate data. This open-access database is available through the DANE website (https://www.dane.gov.co). The birth certificate is completed in the health care facility where the birth occurred and is filled out by the health care professional (doctor or nurse) who delivered the infant. Community health workers and nursing assistants are also authorized to fill out the certificates, particularly in areas with health care provider shortages. According to the United Nations Demographic and Social Statistics and some studies carried out by independent organizations, the coverage and completeness of birth certificates in Colombia have greatly improved in the last years, going from 68% in 2000 to 97% in 2015 [23–25].

Only full-term births were included in the analysis due to the effect of gestational age on the study outcomes and given that the exact gestational age at delivery was not available in the birth certificate dataset, which included gestational age as a categorical variable. This made it impossible to estimate more precise outcomes, such as weight for gestational age. For the study period (2008–2018), 5,859,208 full-term births were identified. The data were also limited to singleton births (n = 5,816,226) and those with complete data on the study outcomes, SEP measures and covariates. All study variables had missing rates lower than 3.2%, and births excluded from the analysis due to missing data made up 6.5% of the initial number of full-term births identified. We preferred a full case analysis over a multiple imputation analysis because of the evidence suggesting a better performance of the former when the missingness is not as high as to jeopardize the validity of the study estimates and their precision [26–28]. The final number of births included in the analysis was 5,433,265. We compared births with complete data that were included in the analysis with those with incomplete data and found significant differences in various study measures (S1 Table). The potential implications of such differences on our results are acknowledged in the Discussion section.

This study was approved by the review board of the Faculty of Medicine from the Universidad Nacional de Colombia (approval letter number 012-121/2020).

### Study variables

We analysed socioeconomic inequalities in three primary outcomes: birth weight, Apgar score and number of prenatal visits. Due to concerns regarding potential identification of individuals, DANE only provides birth weight as a categorical measure in the publicly available birth certificate dataset. We examined binary indicators for low birth weight (<2,500 grams) and an Apgar score <7 at 5 minutes after birth [29]. The number of prenatal visits was treated as a count variable. These outcomes were selected because of their relationship with child health and development and other diverse health outcomes throughout the life course [1–3]. The decision to use information about the Apgar score at 5 minutes after birth was based on the understanding that it provides a better assessment of birth conditions and has shown stronger associations with later outcomes than the Apgar score at 1 minute after birth, the other measure available in the birth certificate [30, 31]. We also examined the number of prenatal visits given the importance of such visits to both neonatal and maternal outcomes [32, 33]. In 2016, the World Health Organization (WHO) recommended at least eight antenatal care contacts to reduce perinatal mortality and improve women’s experience of care [34], and in 2013 the Colombian Ministry of Health guidelines endorsed at least seven prenatal visits [32]. Taking this into account, in sensitivity analysis we used a binary indicator for getting the minimum recommended visits (seven) as an alternative outcome.

Two measures of socioeconomic position were employed: maternal education and health insurance schemes. Maternal education was assessed as the highest education level achieved and categorized into primary school or less, secondary school, technical school, or university. Three categories of health insurance were used: contributory (including exceptional), subsidised, and uninsured. In Colombia, the provision of health care services is based on different health insurance schemes [35]. Each family can be enrolled in one of these schemes based on the working status of their members and household resources. The subsidized scheme comprises those without formal employment who are classified as ‘poor’ based on a proxy means test. The health care provided by the subsidized scheme is mainly funded through tax revenue. In the contributory scheme are those with formal employment or independent jobs who are able to pay a monthly fee. As a special form of the contributory scheme, the exceptional scheme includes petroleum industry workers, armed forces members, and teachers in the public sector, among others. This leaves a proportion of the population (6% in our data) as uninsured, i.e., those who are not in formal employment or able to pay a monthly fee and are not considered ‘poor’ based on the means test. Although not a direct measure of SEP, health insurance status has been used as a proxy for socioeconomic circumstances in previous analyses of health inequalities in Colombia [36, 37] due to the abovementioned features of the different schemes.

Maternal age, marital status, place of residence (urban/rural), region, child’s gender, the number of children the mother has had, and year of birth were used as control variables. In the analyses, these control variables were included in the regression models using the categories shown in Table 1.

**Table 1:** Characteristics of births analysed for the whole study period (2008–2018).

		n	Percentage
**Total**		5,433,265	100
**Weight at birth (grams)**	less than 2,500	131,424	2.42
	2,500 or more	5,301,841	97.58
**Five-minute apgar score (points)**	7 or more	5,408,366	99.54
	Less than 7	24,899	0.46
**Number of prenatal visits**	Mean	6.421	
	SD	2.47	
**Educational level**	University	607,707	11.18
	Technical	513,399	9.45
	Secondary	3,327,603	61.24
	Primary or less	984,556	18.12
**Insurance scheme**	Contributory/Except.	2,336,907	43.01
	Subsidised	2,762,535	50.84
	Uninsured	333,823	6.14
**Maternal age (years)**	Less than 20	1,209,561	22.26
	20 to 24	1,598,826	29.43
	25 to 35	2,098,206	38.62
	35 to 39	413,799	7.62
	40 or more	112,873	2.08
**Newborns’ gender**	Male	2,778,611	51.14
	Female	2,654,654	48.86
**Location of residence**	Urban	4,307,949	79.29
	Small villages	402,397	7.41
	Rural	722,919	13.31
**Marital state**	Married or in consensual union	4,603,555	84.73
	Divorced or widowed	31,614	0.58
	Single	798,096	14.69
**Region of residence**	Andina	2,943,141	54.17
	Caribe	1,343,610	24.73
	Pacifica	789,237	14.53
	Orinoquia	236,514	4.35
	Amazonia	120,763	2.22
**Number of children (including this one)**	One	2,568,380	47.27
	Two	1,685,201	31.02
	Three	700,498	12.89
	Four	257,309	4.74
	Five or more	221,877	4.08

### Statistical analysis

First, we estimated the prevalence/mean of the outcomes by categories of the SEP indicators and assessed the significance of trends in these outcomes by using the chi square test for trend for the binary outcomes, and ANOVA for number of prenatal visits. Second, the relative index of inequality (RII) and slope index of inequality (SII) were derived to quantify inequalities in relative and absolute terms, respectively [38, 39]. These indices were obtained from regression models where the outcomes were regressed on the SEP indicators, one at a time, while adjusting for all the above-mentioned control variables. For this analysis, RII and SII were calculated using generalized linear models, specifying a binomial distribution (log-binomial regression) with a logarithmic link function for RII and an identity link function for SII [40–42]. One of the advantages of the RII and SII is that they use data on all socioeconomic groups and not just those at the highest and lowest levels. This is a particularly useful property in analyses of temporal trends when the sizes of the socioeconomic groups might change over time. We derived the RII and SII for each combination of outcomes and SEP measures for each year and for the whole study period. Bearing in mind the requirements for the indices, the categories for maternal education and health insurance schemes were transformed into numerical scores scaled from 0 to 1, with values reflecting the cumulative proportion of births among mothers with a higher SEP level. These weighted scores were derived for each year for the yearly analysis, and for the whole period when estimating the global RII and SII. The RII can be interpreted as the prevalence ratio of the outcome between those with the hypothetically worst and best socioeconomic conditions [38, 39]. Values of RII>1 signify a higher prevalence of low birth weight or a lower number of prenatal visits among mothers with worse socioeconomic characteristics. The SII is the absolute difference between the predicted outcomes at the two extremes of the SEP distribution, with values of SII>0 demonstrating inequality. Analyses were also conducted stratifying by place of residence (urban/rural), given the differences between urban and rural areas in aspects that could influence health inequalities such as experiences with the armed conflict, access to health services and community support.

## Results

Among the 5,433,265 full-term singleton births analysed for the whole study period, 2.4% were low birth weight, and 0.5% had an Apgar score <7 at five minutes after birth (Table 1). The mean number of prenatal visits was 6.42 (SD = 2.47). Descriptive statistics of the study variables, including maternal demographic and socioeconomic conditions, mother’s number of previous live births, child’s sex, region, and year of birth, are shown in Table 1. Between 2008 and 2018, there was a slight decrease in the prevalence of low birth weight and having an Apgar score <7, going from 2.8% to 2.1% and from 0.6% to 0.4%, respectively (S2 Table). Our findings also indicate a small increase in the mean number of prenatal visits, from 6.23 in 2008 to 6.49 in 2018. It is worth noting that among the years included in the analysis, better outcomes were observed in 2015, i.e., lower low-birth-weight and Apgar<7 prevalence rates and higher number of prenatal visits, and then higher low-birth-weight prevalence rates and a lower number of prenatal visits were apparent from 2016 to 2018. In fact, after peaking in 2015, the number of prenatal visits decreased over the last years of the study period (S2 Table).

For the period of interest, the prevalence/mean of the outcomes by education and health insurance scheme categories presented a general picture of social gradients in the expected direction, i.e., worse outcomes at successively lower SEP levels (S3 Table). Tests for trends were all significant, thus confirming the existence of these social gradients across the three outcomes and two SEP measures analysed. Uninsured mothers and those with an educational attainment of primary school or less had the lowest number of prenatal visits and showed the highest prevalence rates for low birth weight and for an Apgar score <7 (S3 Table).

**Fig 1:**
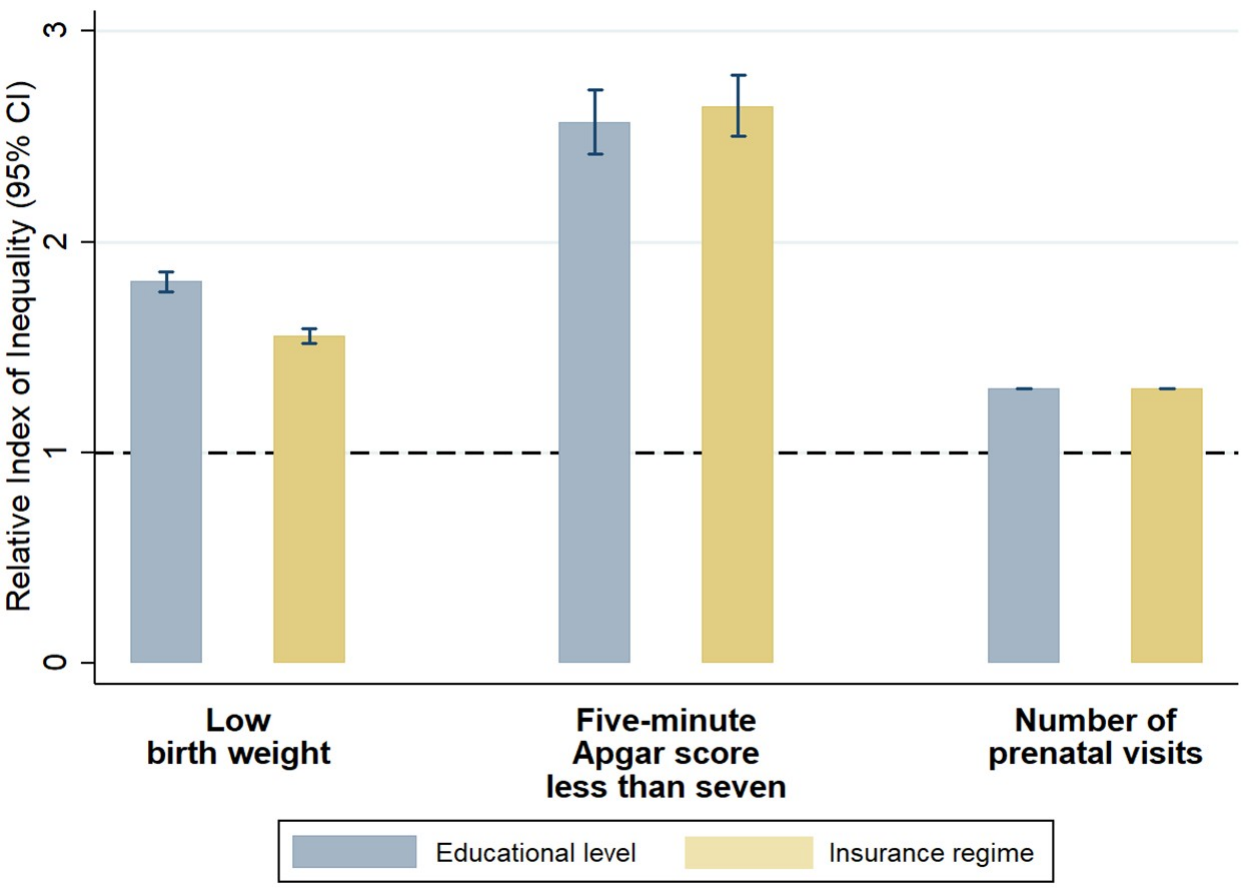
Relative indexes of inequality in birth outcomes and prenatal care by maternal education level and health insurance scheme (including all included births from 2008 to 2018).

**Fig 2:**
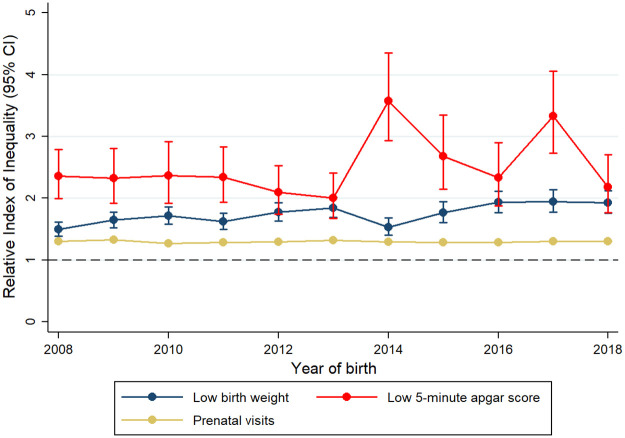
Changes in relative inequalities in birth outcomes and prenatal care by maternal education level from 2008 to 2018.

**Fig 3:**
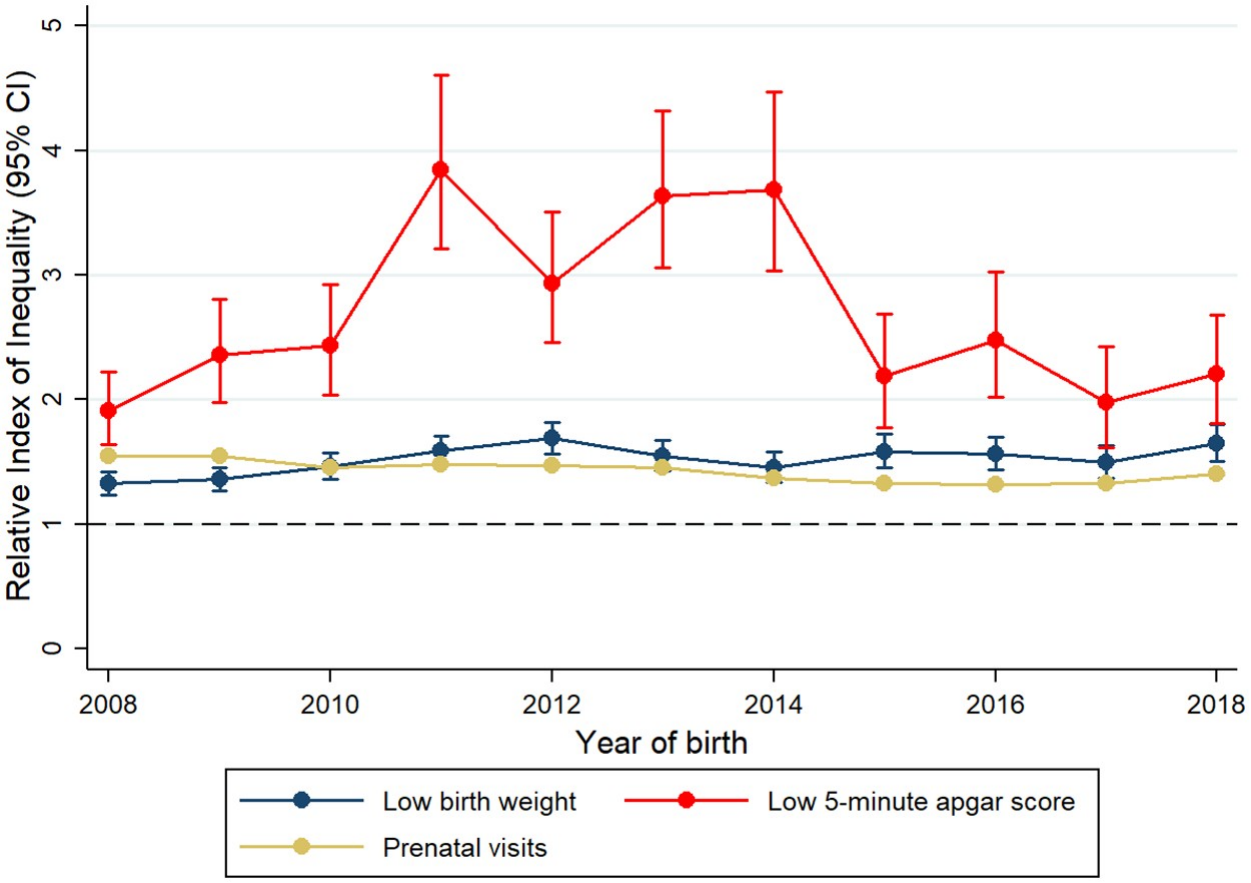
Changes in relative inequalities in birth outcomes and prenatal care by health insurance scheme from 2008 to 2018.

RII and SII estimates showed significant relative and absolute inequalities across all years included in the analysis, all outcomes and all SEP indicators (Figs 1–3, Table 2 and S4 and S5 Tables). For the whole study period, there were larger relative inequalities in having an Apgar score <7 by both education and health insurance scheme (RII: 2.57, 95% CI: 2.42, 2.72 and RII: 2.64, 95% CI: 2.50, 2.79, respectively) (Figs 2 and 3, S4 and S5 Tables). In absolute terms, educational and health insurance-related inequalities were larger for low birth weight, with a prevalence that was 14.1 percentage points higher among mothers with the lowest education level (SII: 14.11, 95% CI: 13.49, 14.73) and 10.8 percentage points higher among uninsured mothers (SII: 10.77, 95% CI: 10.19, 11.35) (Table 2). When comparing the magnitude of inequalities between the two SEP indicators, inequalities in low birth weight were higher across educational levels than across health insurance scheme in both relative and absolute terms, while inequalities in the number of prenatal visits and Apgar score <7 were higher across health insurance scheme.

**Table 2:** Slope indexes of inequality in birth outcomes and prenatal care by maternal education and health insurance scheme, 2008–2018.

	Slope Index of Inequality (CI 95%)		
	Low birth weight* (per 1,000 births)	5-minute Apgar score less than 7 (per 1,000 births)	Prenatal visits (number of visits)
Maternal education			
Overall	14.10 (13.48, 14.72)	4.24 (3.96, 4.51)	1.75 (1.74, 1.76)
2008	11.10 (8.95, 13.26)	5.08 (4.06, 6.11)	1.67 (1.64, 1.71)
2009	14.20 (11.98, 16.41)	3.93 (3.02, 4.84)	1.78 (1.75, 1.81)
2010	15.03 (12.76, 17.31)	3.64 (2.72, 4.56)	1.53 (1.50, 1.56)
2011	12.85 (10.68, 15.02)	3.95 (3.04, 4.86)	1.60 (1.57, 1.63)
2012	14.18 (12.10, 16.26)	3.42 (2.53, 4.31)	1.62 (1.59, 1.65)
2013	14.04 (12.01, 16.07)	3.56 (2.61, 4.50)	1.85 (1.82, 1.88)
2014	8.84 (6.95, 10.72)	5.64 (4.72, 6.57)	1.71 (1.68, 1.74)
2015	11.24 (9.36, 13.12)	3.68 (2.83, 4.53)	1.72 (1.69, 1.75)
2016	14.05 (12.09, 16.00)	3.28 (2.41, 4.15)	1.74 (1.71, 1.77)
2017	13.77 (11.84, 15.71)	5.17 (4.28, 6.07)	1.77 (1.74, 1.81)
2018	13.36 (11.41, 15.31)	3.06 (2.21, 3.91)	1.78 (1.74, 1.81)
Health insurance scheme			
Overall	10.77 (10.19, 11.35)	4.29 (4.04, 4.54)	2.34 (2.34, 2.35)
2008	7.91 (5.91, 9.92)	3.76 (2.85, 4.67)	2.72 (2.69, 2.74)
2009	9.00 (6.96, 11.04)	3.90 (3.07, 4.73)	2.75 (2.72, 2.78)
2010	10.93 (8.86, 13.00)	3.75 (2.97, 4.54)	2.36 (2.33, 2.39)
2011	12.66 (10.65, 14.68)	6.07 (5.23, 6.91)	2.44 (2.41, 2.46)
2012	13.33 (11.41, 15.25)	4.81 (4.00, 5.62)	2.44 (2.41, 2.46)
2013	10.26 (8.35, 12.16)	6.29 (5.43, 7.15)	2.47 (2.44, 2.50)
2014	7.96 (6.18, 9.74)	5.42 (4.60, 6.25)	2.11 (2.08, 2.14)
2015	9.36 (7.57, 11.14)	2.90 (2.12, 3.67)	1.89 (1.86, 1.92)
2016	9.76 (7.90, 11.61)	3.57 (2.76, 4.38)	1.86 (1.83, 1.89)
2017	8.48 (6.66, 10.31)	2.87 (2.01, 3.73)	1.90 (1.87, 1.93)
2018	10.38 (8.51, 12.24)	3.12 (2.33, 3.91)	2.34 (2.31, 2.37)

*less than 2,500 grams at birth

RII and SII estimates with their corresponding CIs revealed a general picture of no significant changes in inequalities over time, with some particular, time-dependent exceptions. For example, particularly high RII estimates were observed for Apgar score <7 by maternal education in 2014 and 2017, and by health insurance in the years 2011–2014 (Figs 2 and 3 and S4 and S5 Tables). When comparing the initial and final years of our study period (2008 and 2018 respectively), inequalities in low birth weight associated with maternal education significantly increased in relative terms, while those in Apgar score <7 decreased in absolute terms (Fig 2, Table 2 and S5 Table). For number of prenatal visits, inequalities related to maternal education remained stable in relative terms and increased in absolute terms (Fig 2, Table 2 and S5 Table). Similar to the picture observed for inequalities related to education, inequalities related to health insurance scheme oscillated more over time for Apgar scores <7 than for low birth weight and number of prenatal visits. Relative inequalities across health insurance schemes increased for low birth weight but decreased for the number of prenatal visits (Fig 3 and S4 Table). In absolute terms, also a small reduction in inequalities in number of prenatal visits was observed from 2008 to 2018 (Table 2). When the alternative outcome of having <7 prenatal visits was considered, RII and SII estimates showed similarities with the aforementioned results for prenatal care. Educational inequalities did not significantly change over time, with some time-dependent exceptions; and inequalities by health insurance were lower during the last five years of the study period (S6 Table).

Regarding analysis by place of residence, there was not a clear and consistent pattern of inequalities across areas (rural/urban). Although inequalities in low birth weight were higher in urban areas for certain years, that was not consistent across the study period, the two SEP measures or nature of inequalities, i.e., absolute or relative (S7 Table).

## Discussion

In this study, we examined socioeconomic inequalities in birth outcomes among infants born in Colombia between 2008 and 2018. Our results showed that among full-term singleton births, there was a slight improvement in the outcomes analysed i.e., lower low-birth-weight and Apgar<7 prevalence rates and higher number of prenatal visits. Interestingly, better birth outcomes and prenatal care estimates were observed in 2015, followed by higher prevalence rates for low birth weight and Apgar <7 and a lower number of prenatal visits in the last years of the time series. We found significant inequalities throughout the study period in all outcomes and across all SEP indicators, showing that the mother’s type of health insurance and education level are important drivers of inequalities in birth outcomes. Changes over time in the magnitude of those inequalities presented a mixed picture with differences according to the outcome, SEP indicator and whether inequalities were measured in relative or absolute terms. Overall, our analysis did not show consistent improvement, and in fact, some inequalities increased over the 11-year period studied.

Although many different factors could contribute to an explanation of our finding of better outcomes in 2015 (lower low-birth-weight and Apgar<7 prevalence rates and higher number of prenatal visits), we think that a potential explanation has to do with the dynamics of the internal armed conflict in Colombia. Specifically, the signing of the 2016 peace accord between the government and the FARC guerrillas was preceded by a definitive ceasefire in July 2015 that introduced differential changes in the levels of conflict violence across municipalities. This decrease in the intensity of conflict violence significantly improved pregnancy outcomes. The effects were larger for women living in municipalities where the intensity of FARC-related violence was historically greater [43].

Our findings suggest that the mother’s health insurance scheme and education level are important determinants of birth outcomes in the Colombian context. The health insurance scheme does not seem to help address health inequalities, which has also been emphasized in other analyses with Colombian data [36, 44–46]. Systematic inequalities exist, with those uninsured or in the subsidized scheme having higher neonatal mortality rates [47], shorter gastric cancer survival rates [36], longer delays in tuberculosis diagnosis [45], less frequent use of preventive services [48, 49], more barriers to accessing health care when needed [50] and poorer oral health [37]. This evidence, together with our findings, suggests that the Colombian health care system urgently needs to shift towards universal coverage and a structural redesign to address these high levels of fragmentation. In line with previous studies, our findings also support the role of maternal education as a determinant of birth outcomes, particularly of low birth weight [14–16]. The overall improvement in maternal education between 2008 and 2018 could have contributed to the amplification of the inequalities between the two extremes of the spectrum because less educated mothers in 2018 were a smaller, more disadvantaged and more vulnerable social group than those in 2008. Educational inequalities have also been identified as drivers of other health outcomes in Colombia [51, 52], which underscores the importance of social policies aimed at guaranteeing access to quality education up to the highest levels.

The general picture of our findings showing no significant changes in the magnitude of inequalities over the study period, with just some exceptions, could be related to the fact that there have not been policy initiatives aimed to tackle key structural social determinants of maternal and child health in Colombia. Moreover, some significant higher relative inequalities observed for Apgar score<7 in certain years, e.g., 2014 could be a result of faster improvements in this outcome among the better educated mothers and those in the contributory or exceptional health insurance schemes.

This is, to our knowledge, the first study to quantify the magnitude of inequalities in birth outcomes based on socioeconomic differences and to describe changes over time using birth certificate data covering virtually all births in Colombia. However, our findings should be interpreted with some caveats. First, births with incomplete data that were excluded from the analysis were more likely to be from rural areas, to have worse outcomes, and to be for mothers with lower educational levels or who are uninsured or in the subsidized scheme, than births included in the analysis (S1 Table). This may have influenced our findings, as the magnitude of inequalities could be even higher than our reported estimates. As the rate of missing data was higher in the first years of our study period (particularly from 2008 to 2010), inequalities could have been underestimated mainly for those years. In addition, the birth certificate data did not include information on other socioeconomic indicators (occupation, income, etc.) or maternal health, which could help us to further understand the observed inequalities in birth outcomes. Another limitation concerns the birth weight variable that was supplied as ordinal groupings to comply with confidentiality requirements, leading to a loss of precision in measurement. Finally, information bias could be an issue due to the retrospective nature of the study, and some issues related to the birth certificates coverage and completeness. However, as mentioned in the Methods section, various analyses have shown a significant improvement in the quality and coverage of the Colombian birth certificate data in recent years [23–25].

The finding of consistently worse birth outcomes among more disadvantaged groups and the lack of a significant improvement in the magnitude of inequalities over an 11-year period is a particularly worrying issue because it could aggravate the cycle of inequality. This is, children born in socioeconomically disadvantaged circumstances are more likely to face difficulties during childhood, teenage years and even adulthood; given the influence of birth outcomes on health, social and economic outcomes throughout the life course. The findings in our analysis emphasize the importance of policies aimed at providing access to quality education and a health care system with universal coverage and high levels of integration. A real commitment to addressing health inequalities in Colombia means that policies and actions should not be focused only on the most disadvantaged but should aim at guaranteeing everyone opportunities and access to material and nonmaterial resources that are important for health (health care, safe environments, etc.). These aims should be part of a governmental and societal commitment to building a fairer society in the Colombian post-conflict era.

## Supporting information

S1 Fig Flowchart illustrating the selection of birth certificates included in the main analysis. (TIF)

S1 Table Characteristics of included and excluded births. (DOCX)

S2 Table Changes in birth outcomes and characteristics of included births from 2008 to 2018. (DOCX)

S3 Table Prevalence (events per 1,000 births) or mean of the outcomes by categories of education and health insurance scheme, 2008–2018. (DOCX)

S4 Table Relative indexes of inequality in birth outcomes and prenatal care by health insurance scheme, 2008–2018. (DOCX)

S5 Table Relative indexes of inequality in birth outcomes and prenatal care by educational level, 2008–2018. (DOCX)

S6 Table Results for the alternative outcome of having less than seven prenatal care visits. (DOCX)

S7 Table Relative and slope indexes of inequality in birth outcomes and prenatal care by two SEP measures and place of residence, 2008–2018. (DOCX)
