# Supplementary figures and images for: Socioeconomic inequalities in birth outcomes: An 11-year analysis in Colombia
Source: PLoS One. 2021 Jul 29;16(7):e0255150. doi: 10.1371/journal.pone.0255150 (PMC8321228; doi:10.1371/journal.pone.0255150)

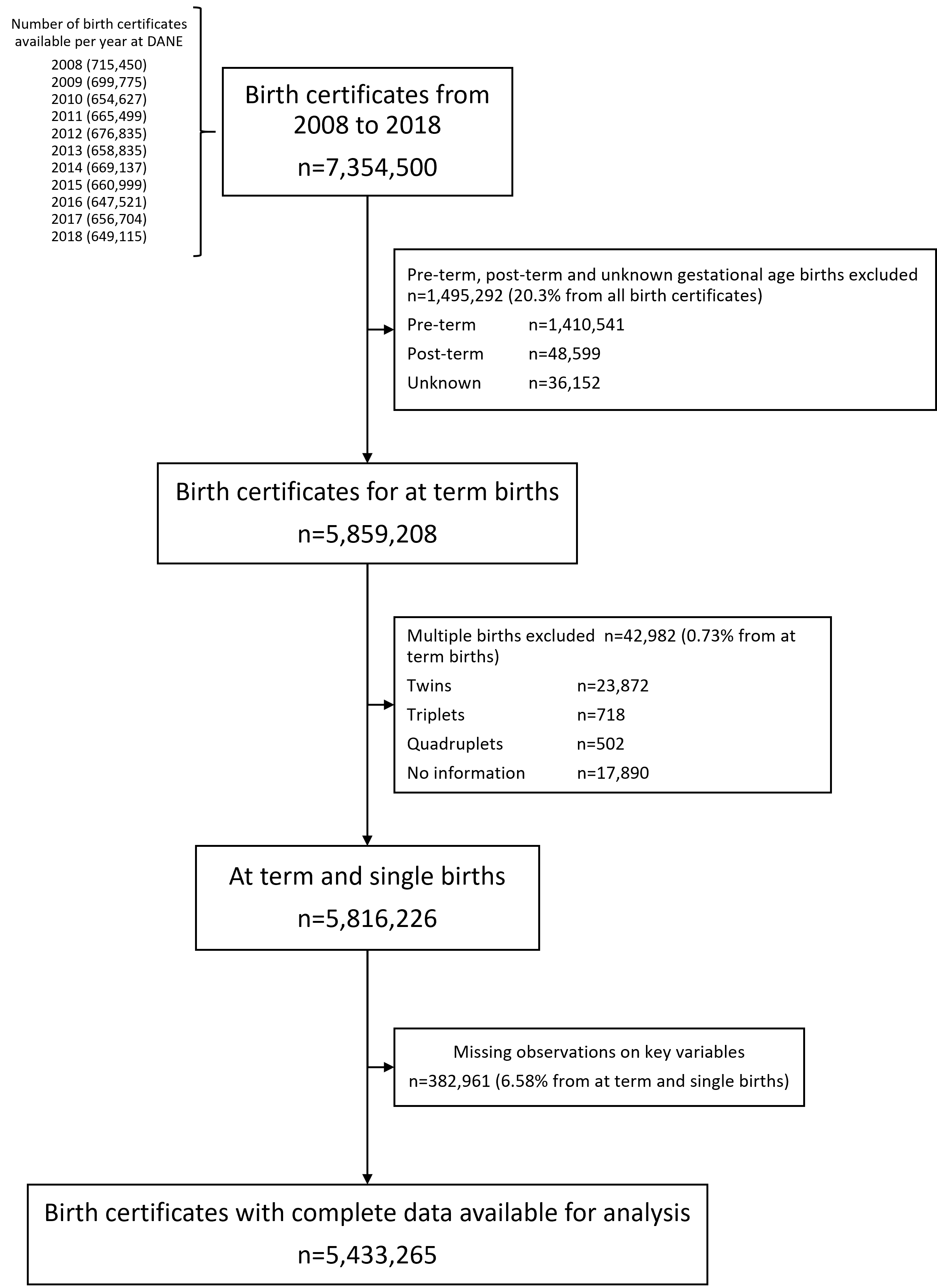

Supplement: S1 Fig — (TIF) [file pone.0255150.s001.tif]
