# Supplementary material for: Socioeconomic inequalities in birth outcomes: An 11-year analysis in Colombia
Source: PLoS One. 2021 Jul 29;16(7):e0255150. doi: 10.1371/journal.pone.0255150 (PMC8321228; doi:10.1371/journal.pone.0255150)
Supplement: S1 Table — (DOCX) [file pone.0255150.s002.docx]

**S1 Table. Characteristics of included and excluded births**

| **Variables** | | **Percentage or mean** | | **Significance level of the difference (p value)^*^** |
| --- | --- | --- | --- | --- |
|  |  | **Individuals included in the analysis with complete data**  (n=5,433,265) | **Individuals excluded from the analysis because of missing values**  (n=382,961) |  |
| **Weight at birth (grams)** | Less than 2,500 | 2.42 | 2.91 |  |
|  | 2,500 or more | 97.58 | 97.09 |  |
|  |  |  |  | <0.0001 |
| **Five-minute apgar score (points)** | 7 or more | 99.54 | 99.16 |  |
|  | Less than 7 | 0.46 | 0.84 |  |
|  |  |  |  | <0.0001 |
| **Number of prenatal visits** | Mean | 6.42 | 5.41 |  |
|  | SD | 2.47 | 2.60 |  |
|  |  |  |  | <0.0001 |
| **Health insurance scheme** | Contributory/Except. | 43.01 | 28.75 |  |
|  | Subsidised | 50.84 | 60.90 |  |
|  | Uninsured | 6.14 | 10.35 |  |
|  |  |  |  | <0.0001 |
| **Educational level** | University | 11.18 | 11.59 |  |
|  | Technical | 9.45 | 5.55 |  |
|  | Secondary | 61.24 | 52.22 |  |
|  | Primary or less | 18.12 | 30.63 |  |
|  |  |  |  | <0.0001 |
| **Region of residence** | Andina | 54.17 | 38.34 |  |
|  | Caribe | 24.73 | 40.55 |  |
|  | Pacífico | 14.53 | 13.19 |  |
|  | Orinoquía | 4.35 | 3.91 |  |
|  | Amazonía | 2.22 | 4.00 |  |
|  |  |  |  | <0.0001 |
| **Newborns' gender** | Female | 51.14 | 51.42 |  |
|  | Male | 48.86 | 48.58 |  |
|  |  |  |  | <0.0001 |
| **Birth year** | 2008 | 9.21 | 14.61 |  |
|  | 2009 | 9.18 | 13.14 |  |
|  | 2010 | 8.60 | 13.53 |  |
|  | 2011 | 9.07 | 10.35 |  |
|  | 2012 | 9.23 | 10.07 |  |
|  | 2013 | 9.23 | 5.83 |  |
|  | 2014 | 9.35 | 6.20 |  |
|  | 2015 | 9.19 | 6.75 |  |
|  | 2016 | 8.99 | 5.99 |  |
|  | 2017 | 9.09 | 6.46 |  |
|  | 2018 | 8.86 | 7.08 |  |
|  |  |  |  | <0.0001 |
| **Maternal age (years)** | Less than 20 | 22.26 | 23.52 |  |
|  | 20 to 24 | 29.43 | 29.01 |  |
|  | 25 to 35 | 38.62 | 37.22 |  |
|  | 35 to 39 | 7.62 | 7.75 |  |
|  | 40 or more | 2.08 | 2.49 |  |
|  |  |  |  | <0.0001 |
| **Location of residence** | Urban | 79.29 | 74.83 |  |
|  | Small villages | 7.41 | 8.45 |  |
|  | Rural | 13.31 | 16.72 |  |
|  |  |  |  | <0.0001 |
| **Marital state** | Married/consensual union | 84.73 | 85.70 |  |
|  | Divorced or widowed | 0.58 | 0.82 |  |
|  | Single | 14.69 | 13.48 |  |
|  |  |  |  | <0.0001 |
| **Number of children (including this one)** | One | 47.27 | 48.54 |  |
|  | Two | 31.02 | 25.39 |  |
|  | Three | 12.89 | 12.94 |  |
|  | Four | 4.74 | 5.96 |  |
|  | Five or more | 4.08 | 7.16 |  |
|  |  |  |  | <0.0001 |

* Chi-squared or t test p-value
