# Supplementary material for: Socioeconomic inequalities in birth outcomes: An 11-year analysis in Colombia
Source: PLoS One. 2021 Jul 29;16(7):e0255150. doi: 10.1371/journal.pone.0255150 (PMC8321228; doi:10.1371/journal.pone.0255150)
Supplement: S2 Table — (DOCX) [file pone.0255150.s003.docx]

**S2 Table. Changes in birth outcomes and characteristics of included births from 2008 to 2018**

|  | **Percentage or mean** | | | | | | | | | | |
| --- | --- | --- | --- | --- | --- | --- | --- | --- | --- | --- | --- |
|  | **2008**  (n=500,196) | **2009**  (n=498,855) | **2010**  (n=467,381) | **2011**  (n=492,939) | **2012**  (n=501,384) | **2013**  (n=501,351) | **2014**  (n=508,195) | **2015**  (n=499,061) | **2016**  (n=488,544) | **2017**  (n=494,004) | **2018**  (n=481,355) |
| **Weight at birth (grams)** |  |  |  |  |  |  |  |  |  |  |  |
| Less than 2,500 | 2.79 | 2.89 | 2.83 | 2.69 | 2.52 | 2.34 | 2.12 | 2.04 | 2.19 | 2.12 | 2.08 |
| 2,500 or more | 97.21 | 97.11 | 97.17 | 97.31 | 97.48 | 97.66 | 97.88 | 97.96 | 97.81 | 97.88 | 97.92 |
| **Five-minute apgar score (points)** |  |  |  |  |  |  |  |  |  |  |  |
| 7 or more | 99.36 | 99.50 | 99.55 | 99.49 | 99.48 | 99.46 | 99.52 | 99.61 | 99.58 | 99.57 | 99.60 |
| Less than 7 | 0.64 | 0.50 | 0.45 | 0.51 | 0.52 | 0.54 | 0.48 | 0.39 | 0.42 | 0.43 | 0.40 |
| **Number of prenatal visits** |  |  |  |  |  |  |  |  |  |  |  |
| Mean | 6.23 | 6.23 | 6.29 | 6.17 | 6.18 | 6.49 | 6.60 | 6.68 | 6.67 | 6.58 | 6.49 |
| SD | 2.6 | 2.6 | 2.3 | 2.4 | 2.3 | 2.5 | 2.4 | 2.5 | 2.5 | 2.5 | 2.6 |
| **Educational level** |  |  |  |  |  |  |  |  |  |  |  |
| University | 10.55 | 10.08 | 10.61 | 10.35 | 10.32 | 10.85 | 11.44 | 12.38 | 12.36 | 12.17 | 12.31 |
| Technical | 6.05 | 7.07 | 7.46 | 7.60 | 8.08 | 8.91 | 9.78 | 11.07 | 11.84 | 12.40 | 12.85 |
| Secondary | 58.46 | 59.61 | 60.08 | 61.11 | 62.22 | 62.21 | 62.07 | 61.38 | 61.26 | 61.15 | 60.95 |
| Primary or less | 24.94 | 23.24 | 21.85 | 20.94 | 19.39 | 18.03 | 16.72 | 15.17 | 14.54 | 14.28 | 13.89 |
| **Health insurance scheme** |  |  |  |  |  |  |  |  |  |  |  |
| Contributory/Except. | 40.35 | 39.98 | 41.14 | 41.38 | 41.55 | 41.83 | 42.80 | 45.57 | 45.77 | 42.92 | 40.22 |
| Subsidised | 46.71 | 48.49 | 48.01 | 50.56 | 52.56 | 53.60 | 53.91 | 51.95 | 52.00 | 54.53 | 54.21 |
| Uninsured | 12.94 | 11.54 | 10.85 | 8.06 | 5.89 | 4.57 | 3.29 | 2.49 | 2.22 | 2.55 | 5.58 |
| **Maternal age (years)** |  |  |  |  |  |  |  |  |  |  |  |
| Less than 19 | 23.33 | 23.36 | 23.38 | 23.41 | 23.89 | 23.40 | 22.40 | 21.33 | 20.81 | 20.43 | 19.78 |
| 20 to 24 | 29.50 | 29.52 | 29.34 | 29.24 | 29.54 | 29.61 | 29.64 | 29.14 | 29.44 | 29.31 | 29.11 |
| 25 to 35 | 37.49 | 37.70 | 37.95 | 37.94 | 37.40 | 37.73 | 38.43 | 39.52 | 39.64 | 39.75 | 40.46 |
| 35 to 39 | 7.44 | 7.23 | 7.18 | 7.25 | 7.06 | 7.23 | 7.56 | 7.99 | 8.10 | 8.44 | 8.48 |
| 40 or more | 2.24 | 2.19 | 2.15 | 2.16 | 2.10 | 2.04 | 1.97 | 2.03 | 2.02 | 2.07 | 2.16 |
| **Location of residence** |  |  |  |  |  |  |  |  |  |  |  |
| Urban | 79.58 | 79.44 | 79.45 | 78.36 | 78.31 | 78.57 | 79.32 | 79.66 | 79.55 | 78.55 | 78.40 |
| Small villages | 7.62 | 7.84 | 6.90 | 7.39 | 7.66 | 7.49 | 7.33 | 7.27 | 7.38 | 7.78 | 7.47 |
| Rural | 12.80 | 12.72 | 13.65 | 14.25 | 14.03 | 13.94 | 13.35 | 13.08 | 13.07 | 13.66 | 14.14 |
| **Marital state** |  |  |  |  |  |  |  |  |  |  |  |
| Married or in consensual union | 83.86 | 84.01 | 83.78 | 84.35 | 84.41 | 84.51 | 85.09 | 85.28 | 85.54 | 85.91 | 85.96 |
| Divorced or widowed | 1.01 | 0.91 | 0.74 | 0.64 | 0.55 | 0.54 | 0.49 | 0.42 | 0.39 | 0.39 | 0.40 |
| Single | 15.13 | 15.08 | 15.48 | 15.01 | 15.05 | 14.95 | 14.42 | 14.30 | 14.07 | 13.70 | 13.64 |
| **Number of children (including this one)** |  |  |  |  |  |  |  |  |  |  |  |
| One | 44.25 | 44.61 | 45.89 | 47.26 | 48.61 | 48.65 | 48.61 | 48.69 | 48.88 | 48.47 | 47.22 |
| Two | 30.13 | 30.23 | 30.20 | 29.55 | 29.37 | 29.91 | 30.76 | 31.31 | 31.58 | 31.90 | 32.45 |
| Three | 14.38 | 14.18 | 13.67 | 13.20 | 12.67 | 12.53 | 12.19 | 12.10 | 12.01 | 12.14 | 12.64 |
| Four | 5.82 | 5.73 | 5.39 | 5.23 | 4.90 | 4.67 | 4.47 | 4.21 | 4.07 | 4.11 | 4.25 |
| Five or more | 5.43 | 5.25 | 4.86 | 4.77 | 4.45 | 4.24 | 3.97 | 3.68 | 3.47 | 3.38 | 3.45 |
| **Region of residence** |  |  |  |  |  |  |  |  |  |  |  |
| Andina | 55.52 | 54.78 | 55.24 | 53.39 | 52.71 | 52.83 | 52.50 | 52.77 | 52.74 | 51.26 | 50.37 |
| Caribe | 23.48 | 24.39 | 23.34 | 25.27 | 26.02 | 25.96 | 26.32 | 26.15 | 26.39 | 27.60 | 28.86 |
| Pacifica | 14.67 | 14.59 | 14.80 | 14.70 | 14.56 | 14.47 | 14.49 | 14.23 | 14.20 | 14.28 | 13.77 |
| Orinoquia | 4.05 | 3.93 | 4.30 | 4.25 | 4.40 | 4.43 | 4.43 | 4.53 | 4.38 | 4.40 | 4.50 |
| Amazonia | 2.29 | 2.31 | 2.32 | 2.38 | 2.31 | 2.32 | 2.27 | 2.32 | 2.29 | 2.46 | 2.49 |
