# Supplementary material for: Socioeconomic inequalities in birth outcomes: An 11-year analysis in Colombia
Source: PLoS One. 2021 Jul 29;16(7):e0255150. doi: 10.1371/journal.pone.0255150 (PMC8321228; doi:10.1371/journal.pone.0255150)
Supplement: S3 Table — (DOCX) [file pone.0255150.s004.docx]

**S3 Table. Prevalence (events per 1,000 births) or mean of the outcomes by categories of education and health insurance scheme, 2008-2018**

|  | **Total population**  **per category** | **Low birth weight** | | **Five-minute Apgar score less than 7** | | **Number of pre-natal visits [mean (SD)]** | |
| --- | --- | --- | --- | --- | --- | --- | --- |
| **Educational level** |  |  |  |  |  |  |  |
| University | 607,707 | 19.35 |  | 2.85 |  | 7.93 (2.3) |  |
| Technical | 513,399 | 20.48 |  | 3.42 |  | 7.34 (2.1) |  |
| Secondary | 3,327,603 | 24.83 |  | 4.63 |  | 6.32 (2.3) |  |
| Primary or less | 984,556 | 26.96 | p<0.001^*^ | 6.08 | p<0.001^*^ | 5.36 (2.6) | p<0.001^**^ |
| **Health Insurance Scheme** |  |  |  |  |  |  |  |
| Contributory/Exceptional | 2,336,907 | 22.04 |  | 3.24 |  | 7.35 (2.2) |  |
| Subsidised | 2,762,535 | 25.05 |  | 5.47 |  | 5.83 (2.4) |  |
| Uninsured | 333,823 | 32.10 | p<0.001^*^ | 6.60 | p<0.001^*^ | 4.78 (2.66) | p<0.001^**^ |

* Chi-squared p-value for the trend

** ANOVA p value for differences from the previous level

Note: hypothesis testing suggests an increasing prevalence of low birth weight and low five-minute Apgar score and decreasing numbers of pre-natal visits with decreasing levels of education and increasing limitations of access to health care (represented by changes from contributory to subsidised insurance schemes and from subsided to uninsured).
