# Supplementary material for: Socioeconomic inequalities in birth outcomes: An 11-year analysis in Colombia
Source: PLoS One. 2021 Jul 29;16(7):e0255150. doi: 10.1371/journal.pone.0255150 (PMC8321228; doi:10.1371/journal.pone.0255150)
Supplement: S4 Table — (DOCX) [file pone.0255150.s005.docx]

**S4 Table. Relative Indexes of Inequality in birth outcomes and prenatal care by health insurance scheme, 2008-2018**

|  | **Relative Index of Inequality (IC 95%)** | | |
| --- | --- | --- | --- |
|  | **Low birth weigth*** | **5-minute Apgar score less than 7** | **Prenatal visits** |
| Overall | 1.55 (1.52, 1.59) | 2.64 (2.50, 2.79) | 1.43 (1.43, 1.43) |
| 2008 | 1.32 (1.23, 1.42) | 1.91 (1.64, 2.22) | 1.54 (1.54, 1.55) |
| 2009 | 1.36 (1.27, 1.46) | 2.35 (1.98, 2.81) | 1.55 (1.54, 1.55) |
| 2010 | 1.46 (1.36, 1.57) | 2.44 (2.03, 2.92) | 1.45 (1.45, 1.46) |
| 2011 | 1.59 (1.48, 1.71) | 3.85 (3.21, 4.61) | 1.48 (1.47, 1.48) |
| 2012 | 1.69 (1.57, 1.82) | 2.93 (2.46, 3.50) | 1.47 (1.47, 1.48) |
| 2013 | 1.55 (1.43, 1.67) | 3.64 (3.06, 4.32) | 1.45 (1.45, 1.46) |
| 2014 | 1.45 (1.34, 1.58) | 3.68 (3.04, 4.47) | 1.37 (1.36, 1.37) |
| 2015 | 1.58 (1.45, 1.72) | 2.19 (1.78, 2.69) | 1.32 (1.32, 1.33) |
| 2016 | 1.56 (1.44, 1.70) | 2.47 (2.02, 3.02) | 1.31 (1.31, 1.32) |
| 2017 | 1.49 (1.37, 1.63) | 1.98 (1.62, 2.42) | 1.32 (1.32, 1.33) |
| 2018 | 1.64 (1.51, 1.80) | 2.20 (1.81, 2.68) | 1.40 (1.40, 1.41) |

*less than 2,500 grams at birth
