# Supplementary material for: Socioeconomic inequalities in birth outcomes: An 11-year analysis in Colombia
Source: PLoS One. 2021 Jul 29;16(7):e0255150. doi: 10.1371/journal.pone.0255150 (PMC8321228; doi:10.1371/journal.pone.0255150)
Supplement: S5 Table — (DOCX) [file pone.0255150.s006.docx]

**S5 Table. Relative Indexes of Inequality in birth outcomes and prenatal care by educational level, 2008-2018**

|  | **Relative Index of Inequality (IC 95%)** | | |
| --- | --- | --- | --- |
|  | **Low birth weight*** | **5-minute Apgar score less than 7** | **Prenatal visits** |
| Overall | 1.81 (1.77, 1.86) | 2.57 (2.42, 2.72) | 1.30 (1.30, 1.31) |
| 2008 | 1.49 (1.38, 1.61) | 2.36 (1.99, 2.79) | 1.30 (1.30, 1.31) |
| 2009 | 1.64 (1.52, 1.77) | 2.32 (1.92, 2.80) | 1.32 (1.32, 1.33) |
| 2010 | 1.71 (1.58, 1.86) | 2.36 (1.91, 2.92) | 1.27 (1.26, 1.28) |
| 2011 | 1.62 (1.49, 1.75) | 2.34 (1.93, 2.83) | 1.29 (1.28, 1.29) |
| 2012 | 1.78 (1.63, 1.93) | 2.10 (1.74, 2.53) | 1.29 (1.29, 1.30) |
| 2013 | 1.84 (1.69, 2.01) | 2.01 (1.67, 2.40) | 1.32 (1.32, 1.33) |
| 2014 | 1.53 (1.40, 1.68) | 3.57 (2.93, 4.35) | 1.29 (1.28, 1.29) |
| 2015 | 1.77 (1.61, 1.94) | 2.68 (2.14, 3.35) | 1.28 (1.28, 1.29) |
| 2016 | 1.93 (1.77, 2.12) | 2.33 (1.88, 2.90) | 1.29 (1.28, 1.29) |
| 2017 | 1.95 (1.78, 2.13) | 3.33 (2.73, 4.06) | 1.30 (1.29, 1.31) |
| 2018 | 1.93 (1.76, 2.12) | 2.18 (1.76, 2.70) | 1.30 (1.30, 1.31) |

*less than 2,500 grams at birth
