# Supplementary material for: Socioeconomic inequalities in birth outcomes: An 11-year analysis in Colombia
Source: PLoS One. 2021 Jul 29;16(7):e0255150. doi: 10.1371/journal.pone.0255150 (PMC8321228; doi:10.1371/journal.pone.0255150)
Supplement: S6 Table — (DOCX) [file pone.0255150.s007.docx]

**S6 Table A. Prevalence of having less than seven prenatal care visits by categories of education and health insurance scheme, 2008-2018**

|  | **Total population per category** | **Less than 7 visits (%)** | |
| --- | --- | --- | --- |
| **Educational level** |  |  | |
| University | 607,707 | 23.99 |  |
| Technical | 513,399 | 31.35 |  |
| Secondary | 3,327,603 | 49.76 |  |
| Primary or less | 984,556 | 63.98 | p<0.001^*^ |
| **Health Insurance Scheme** |  |  |  |
| Contributory/Exceptional | 2,336,907 | 32.30 |  |
| Subsidised | 2,762,535 | 57.78 |  |
| Uninsured | 333,823 | 72.32 | p<0.001^*^ |

* Chi-squared p-value for the trend

**S6 Table B Relative and Slope Indexes of Inequality for having less than seven prenatal care visits (percentage) by maternal education and health insurance scheme, 2008-2018**

|  | Relative Index of Inequality (CI 95%) | | Slope Index of Inequality (CI 95%) | |
| --- | --- | --- | --- | --- |
|  | Maternal education | Health insurance scheme | Maternal education | Health insurance scheme |
| Overall | 1.64 (1.63, 1.64) | 2.10 (2.10, 2.11) | 27.48 (27.30, 27.67) | 38.72 (38.54, 38.90) |
| 2008 | 1.62 (1.60, 1.64) | 2.51 (2.48, 2.54) | 24.79 (24.18, 25.41) | 43.75 (43.19, 44.31) |
| 2009 | 1.63 (1.61, 1.66) | 2.47 (2.44, 2.50) | 25.43 (24.81, 26.05) | 43.15 (42.60, 43.71) |
| 2010 | 1.61 (1.59, 1.63) | 2.28 (2.25, 2.31) | 25.34 (24.69, 25.99) | 40.61 (40.03, 41.19) |
| 2011 | 1.68 (1.66, 1.70) | 2.34 (2.31, 2.37) | 27.00 (26.37, 27.63) | 41.39 (40.82, 41.97) |
| 2012 | 1.73 (1.70, 1.75) | 2.38 (2.35, 2.41) | 28.57 (27.94, 29.20) | 42.96 (42.38, 43.54) |
| 2013 | 1.66 (1.64, 1.68) | 2.15 (2.12, 2.17) | 28.82 (28.20, 29.45) | 41.04 (40.46, 41.63) |
| 2014 | 1.56 (1.54, 1.57) | 1.85 (1.83, 1.87) | 26.10 (25.48, 26.72) | 34.53 (33.95, 35.12) |
| 2015 | 1.53 (1.51, 1.55) | 1.74 (1.72, 1.75) | 25.75 (25.12, 26.37) | 31.22 (30.63, 31.82) |
| 2016 | 1.57 (1.55, 1.59) | 1.73 (1.71, 1.75) | 27.14 (26.51, 27.77) | 30.63 (30.03, 31.24) |
| 2017 | 1.61 (1.59, 1.63) | 1.78 (1.76, 1.80) | 27.84 (27.21, 28.47) | 31.98 (31.37, 32.59) |
| 2018 | 1.62 (1.61, 1.64) | 1.97 (1.95, 2.00) | 27.91 (27.27, 28.55) | 36.66 (36.06, 37.26) |
