# Supplementary material for: Socioeconomic inequalities in birth outcomes: An 11-year analysis in Colombia
Source: PLoS One. 2021 Jul 29;16(7):e0255150. doi: 10.1371/journal.pone.0255150 (PMC8321228; doi:10.1371/journal.pone.0255150)
Supplement: S7 Table — (DOCX) [file pone.0255150.s008.docx]

**S7 Table A Relative Indexes of Inequality in birth outcomes and prenatal care by two SEP measures and place of residence, 2008-2018**

|  | Relative Index of Inequality (CI 95%) | | | | | |
| --- | --- | --- | --- | --- | --- | --- |
|  | Urban | | | Rural | | |
|  | Low birth weigth* | 5-minute Apgar score less than 7 | Prenatal visits | Low birth weigth* | 5-minute Apgar score less than 7 | Prenatal visits |
| Maternal education | | | | | | |
| Overall | 1.78 (1.73, 1.83) | 2.60 (2.42, 2.78) | 1.31 (1.31, 1.31) | 1.98 (1.87, 2.10) | 2.38 (2.11, 2.69) | 1.29 (1.29, 1.30) |
| 2008 | 1.47 (1.35, 1.60) | 2.52 (2.08, 3.06) | 1.31 (1.30, 1.32) | 1.55 (1.30, 1.85) | 1.87 (1.33, 2.64) | 1.28 (1.26, 1.29) |
| 2009 | 1.58 (1.45, 1.72) | 2.23 (1.78, 2.79) | 1.33 (1.33, 1.34) | 1.94 (1.63, 2.31) | 2.66 (1.83, 3.86) | 1.28 (1.26, 1.30) |
| 2010 | 1.68 (1.54, 1.84) | 2.45 (1.92, 3.13) | 1.28 (1.27, 1.28) | 1.87 (1.56, 2.24) | 1.84 (1.22, 2.77) | 1.25 (1.24, 1.27) |
| 2011 | 1.65 (1.51, 1.81) | 2.62 (2.09, 3.27) | 1.30 (1.29, 1.31) | 1.48 (1.24, 1.77) | 1.62 (1.12, 2.34) | 1.25 (1.24, 1.27) |
| 2012 | 1.73 (1.58, 1.90) | 2.30 (1.85, 2.85) | 1.30 (1.29, 1.31) | 1.91 (1.60, 2.28) | 1.47 (1.00, 2.14) | 1.27 (1.26, 1.29) |
| 2013 | 1.85 (1.68, 2.04) | 2.35 (1.91, 2.91) | 1.33 (1.32, 1.34) | 1.90 (1.57, 2.30) | 1.09 (0.76, 1.56) | 1.30 (1.29, 1.32) |
| 2014 | 1.51 (1.36, 1.67) | 3.22 (2.55, 4.08) | 1.29 (1.29, 1.30) | 1.68 (1.37, 2.05) | 4.61 (3.14, 6.78) | 1.27 (1.26, 1.28) |
| 2015 | 1.73 (1.55, 1.92) | 2.09 (1.61, 2.71) | 1.29 (1.28, 1.29) | 1.97 (1.58, 2.46) | 5.72 (3.65, 8.97) | 1.27 (1.25, 1.28) |
| 2016 | 1.86 (1.68, 2.06) | 2.55 (1.99, 3.27) | 1.29 (1.28, 1.30) | 2.46 (2.00, 3.02) | 1.68 (1.05, 2.70) | 1.28 (1.27, 1.30) |
| 2017 | 1.93 (1.74, 2.14) | 3.38 (2.71, 4.22) | 1.30 (1.30, 1.31) | 2.05 (1.67, 2.52) | 3.41 (2.15, 5.41) | 1.29 (1.28, 1.31) |
| 2018 | 1.97 (1.77, 2.19) | 2.11 (1.66, 2.68) | 1.31 (1.30, 1.32) | 1.90 (1.53, 2.37) | 2.42 (1.49, 3.92) | 1.28 (1.26, 1.29) |
| Health insurance scheme | | | | | | |
| Overall | 1.58 (1.54, 1.62) | 2.67 (2.52, 2.84) | 1.45 (1.44, 1.45) | 1.42 (1.33, 1.52) | 2.46 (2.12, 2.86) | 1.36 (1.35, 1.36) |
| 2008 | 1.35 (1.26, 1.46) | 1.99 (1.69, 2.35) | 1.55 (1.55, 1.56) | 1.05 (0.86, 1.28) | 1.48 (1.02, 2.16) | 1.49 (1.47, 1.51) |
| 2009 | 1.39 (1.29, 1.49) | 2.29 (1.90, 2.78) | 1.56 (1.55, 1.56) | 1.10 (0.90, 1.33) | 2.80 (1.74, 4.51) | 1.50 (1.48, 1.52) |
| 2010 | 1.51 (1.40, 1.62) | 2.52 (2.06, 3.07) | 1.46 (1.46, 1.47) | 1.21 (1.00, 1.46) | 1.87 (1.19, 2.93) | 1.41 (1.39, 1.42) |
| 2011 | 1.61 (1.49, 1.74) | 3.86 (3.17, 4.69) | 1.49 (1.49, 1.50) | 1.42 (1.16, 1.74) | 4.11 (2.47, 6.84) | 1.40 (1.39, 1.42) |
| 2012 | 1.68 (1.55, 1.82) | 2.97 (2.45, 3.59) | 1.49 (1.48, 1.50) | 1.68 (1.35, 2.09) | 2.72 (1.60, 4.61) | 1.40 (1.38, 1.41) |
| 2013 | 1.55 (1.42, 1.69) | 3.76 (3.12, 4.53) | 1.47 (1.46, 1.48) | 1.57 (1.25, 1.99) | 2.71 (1.67, 4.37) | 1.37 (1.35, 1.38) |
| 2014 | 1.48 (1.35, 1.62) | 3.89 (3.16, 4.79) | 1.39 (1.38, 1.39) | 1.35 (1.07, 1.71) | 3.12 (1.82, 5.34) | 1.28 (1.26, 1.29) |
| 2015 | 1.64 (1.49, 1.79) | 2.19 (1.75, 2.75) | 1.34 (1.34, 1.35) | 1.28 (1.02, 1.62) | 2.42 (1.40, 4.17) | 1.23 (1.21, 1.24) |
| 2016 | 1.60 (1.46, 1.75) | 2.49 (2.00, 3.09) | 1.33 (1.32, 1.34) | 1.50 (1.20, 1.88) | 2.08 (1.26, 3.45) | 1.24 (1.22, 1.25) |
| 2017 | 1.52 (1.39, 1.67) | 1.94 (1.56, 2.42) | 1.34 (1.34, 1.35) | 1.43 (1.14, 1.80) | 2.14 (1.24, 3.70) | 1.25 (1.23, 1.26) |
| 2018 | 1.73 (1.57, 1.90) | 2.30 (1.87, 2.84) | 1.43 (1.42, 1.44) | 1.33 (1.04, 1.69) | 1.59 (0.95, 2.66) | 1.27 (1.26, 1.29) |

**S7 Table B Slope Indexes of Inequality in birth outcomes and prenatal care by two SEP measures and place of residence, 2008-2018**

|  | Slope Index of Inequality (CI 95%) | | | | | |
| --- | --- | --- | --- | --- | --- | --- |
|  | Urban | | | Rural | | |
|  | Low birth weigth*  (percentage) | 5-minute Apgar score less than 7  (percentage) | Prenatal visits  (number of visits) | Low birth weigth*  (percentage) | 5-minute Apgar score less than 7  (percentage) | Prenatal visits  (number of visits) |
| Maternal education | | | | | | |
| Overall | 13.49 (12.80, 14.18) | 4.00 (3.71, 4.30) | 1.80 (1.79, 1.81) | 17.13 (15.69, 18.58) | 5.11 (4.41, 5.82) | 1.57 (1.55, 1.60) |
| 2008 | 10.59 (8.19, 12.99) | 5.17 (4.05, 6.29) | 1.73 (1.70, 1.77) | 12.58 (7.56, 17.61) | 4.77 (2.22, 7.32) | 1.41 (1.34, 1.48) |
| 2009 | 12.94 (10.48, 15.41) | 3.46 (2.47, 4.46) | 1.86 (1.82, 1.89) | 19.42 (14.31, 24.54) | 5.99 (3.73, 8.25) | 1.43 (1.36, 1.50) |
| 2010 | 14.43 (11.88, 16.97) | 3.50 (2.51, 4.49) | 1.59 (1.56, 1.63) | 17.98 (12.80, 23.16) | 3.59 (1.18, 5.99) | 1.33 (1.26, 1.40) |
| 2011 | 13.28 (10.84, 15.71) | 4.19 (3.18, 5.21) | 1.69 (1.65, 1.72) | 10.86 (5.97, 15.74) | 2.97 (0.79, 5.14) | 1.32 (1.25, 1.38) |
| 2012 | 13.39 (11.05, 15.73) | 3.61 (2.65, 4.58) | 1.69 (1.66, 1.72) | 16.94 (12.29, 21.59) | 2.34 (0.11, 4.57) | 1.40 (1.33, 1.47) |
| 2013 | 13.99 (11.72, 16.26) | 4.05 (3.04, 5.07) | 1.92 (1.88, 1.95) | 15.74 (11.07, 20.41) | 0.73 (-1.71, 3.18) | 1.60 (1.53, 1.67) |
| 2014 | 8.34 (6.27, 10.41) | 4.59 (3.63, 5.55) | 1.76 (1.73, 1.79) | 11.73 (7.13, 16.32) | 10.46 (7.79, 13.14) | 1.51 (1.44, 1.58) |
| 2015 | 10.61 (8.55, 12.67) | 2.49 (1.60, 3.38) | 1.76 (1.73, 1.80) | 14.55 (9.82, 19.28) | 9.17 (6.76, 11.59) | 1.54 (1.47, 1.62) |
| 2016 | 12.92 (10.76, 15.07) | 3.39 (2.48, 4.31) | 1.78 (1.74, 1.81) | 21.13 (16.28, 25.98) | 2.70 (0.21, 5.18) | 1.61 (1.53, 1.68) |
| 2017 | 13.34 (11.20, 15.48) | 5.15 (4.16, 6.13) | 1.82 (1.78, 1.85) | 16.18 (11.52, 20.84) | 5.73 (3.53, 7.93) | 1.64 (1.57, 1.71) |
| 2018 | 13.64 (11.47, 15.81) | 2.86 (1.92, 3.81) | 1.82 (1.79, 1.86) | 13.80 (9.17, 18.43) | 3.78 (1.72, 5.84) | 1.60 (1.52, 1.67) |
| Health insurance scheme | | | | | | |
| Overall | 11.15 (10.52, 11.78) | 4.20 (3.94, 4.47) | 2.41 (2.40, 2.42) | 8.63 (7.07, 10.19) | 4.79 (4.06, 5.53) | 2.00 (1.98, 2.02) |
| 2008 | 8.63 (6.46, 10.79) | 3.85 (2.89, 4.81) | 2.77 (2.74, 2.79) | 1.29 (-4.29, 6.88) | 2.94 (0.21, 5.67) | 2.43 (2.35, 2.51) |
| 2009 | 9.72 (7.52, 11.92) | 3.64 (2.77, 4.51) | 2.80 (2.77, 2.83) | 2.68 (-2.98, 8.34) | 5.92 (3.30, 8.53) | 2.50 (2.42, 2.58) |
| 2010 | 11.82 (9.57, 14.06) | 3.69 (2.87, 4.52) | 2.42 (2.39, 2.44) | 5.38 (-0.06, 10.82) | 3.51 (1.05, 5.96) | 2.12 (2.04, 2.20) |
| 2011 | 13.10 (10.91, 15.29) | 5.87 (4.97, 6.77) | 2.50 (2.47, 2.53) | 9.53 (4.21, 14.86) | 7.45 (5.00, 9.89) | 2.12 (2.04, 2.19) |
| 2012 | 13.33 (11.25, 15.42) | 4.77 (3.91, 5.63) | 2.50 (2.47, 2.53) | 12.66 (7.59, 17.73) | 5.05 (2.67, 7.43) | 2.12 (2.05, 2.19) |
| 2013 | 10.33 (8.28, 12.39) | 6.27 (5.35, 7.19) | 2.54 (2.51, 2.58) | 10.55 (5.40, 15.69) | 5.97 (3.44, 8.51) | 2.04 (1.96, 2.12) |
| 2014 | 8.27 (6.35, 10.19) | 5.39 (4.52, 6.26) | 2.19 (2.16, 2.22) | 6.74 (1.70, 11.77) | 6.36 (3.79, 8.94) | 1.62 (1.54, 1.70) |
| 2015 | 10.05 (8.11, 11.98) | 2.75 (1.94, 3.57) | 1.99 (1.95, 2.02) | 5.27 (0.48, 10.06) | 4.21 (1.88, 6.53) | 1.38 (1.30, 1.46) |
| 2016 | 10.16 (8.15, 12.18) | 3.46 (2.60, 4.33) | 1.95 (1.92, 1.98) | 9.26 (4.38, 14.15) | 3.67 (1.33, 6.02) | 1.44 (1.36, 1.51) |
| 2017 | 8.79 (6.81, 10.78) | 2.77 (1.84, 3.70) | 1.99 (1.96, 2.03) | 7.92 (3.11, 12.73) | 3.34 (1.12, 5.56) | 1.49 (1.41, 1.57) |
| 2018 | 11.39 (9.37, 13.41) | 3.29 (2.43, 4.15) | 2.46 (2.43, 2.49) | 6.03 (1.06, 11.00) | 1.95 (-0.10, 4.00) | 1.74 (1.66, 1.83) |
